# 3-Hydr­oxy-4-nitro­phenyl acetate

**DOI:** 10.1107/S1600536808040981

**Published:** 2008-12-10

**Authors:** Chao Liu, Chunqing Cheng, Xiujie Ji

**Affiliations:** a School of Materials Science and Engineering, Hebei University of Technology, Tianjin 300130, People’s Republic of China; b School of Pharmacy, Jiangxi Science and Technology, Normal University, Jiangxi 330013, People’s Republic of China; c Tianjin Municipal Key Laboratory of Fiber Modification and Functional Fibers, Tianjin Polytechnic University, Tianjin 300160, People’s Republic of China

## Abstract

In the mol­ecule of the title compound, C_8_H_7_NO_5_, the acetate group is oriented with respect to the aromatic ring at a dihedral angle of 85.30 (3)°. An intra­molecular O—H⋯O hydrogen bond results in the formation of a non-planar six-membered ring, adopting an envelope conformation. In the crystal structure, inter­molecular C—H⋯O hydrogen bonds link the mol­ecules.

## Related literature

For general background to phenolic esters as inter­mediates in organic synthesis, see: Trollsås *et al.* (1996 ▶); Svensson *et al.* (1998 ▶); Atkinson *et al.* (2005 ▶); Hu *et al.* (2001 ▶). For a related structure, see: Ji *et al.* (2006 ▶). For bond-length data, see: Allen *et al.* (1987 ▶).
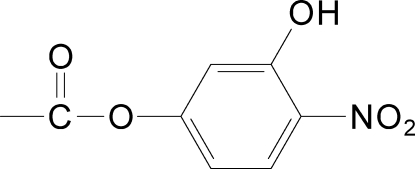


## Experimental

### 

#### Crystal data

C_8_H_7_NO_5_
*M* *_r_* = 197.15
Monoclinic, 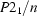

*a* = 10.881 (2) Å
*b* = 5.0543 (10) Å
*c* = 15.318 (3) Å
β = 93.75 (3)°
*V* = 840.6 (3) Å^3^
*Z* = 4
Mo *K*α radiation
μ = 0.13 mm^−1^
*T* = 153 (2) K
0.24 × 0.20 × 0.16 mm

#### Data collection

Bruker SMART diffractometer
Absorption correction: multi-scan (*SADABS*; Sheldrick, 1996 ▶) *T*_min_ = 0.969, *T*_max_ = 0.979
5058 measured reflections
1449 independent reflections
1232 reflections with *I* > 2σ(*I*)
*R*_int_ = 0.027

#### Refinement

*R*[*F* ^2^ > 2σ(*F* ^2^)] = 0.028
*wR*(*F* ^2^) = 0.083
*S* = 1.12
1449 reflections
129 parameters
H-atom parameters constrained
Δρ_max_ = 0.18 e Å^−3^
Δρ_min_ = −0.20 e Å^−3^

### 

Data collection: *SMART* (Bruker, 1997 ▶); cell refinement: *SAINT* (Bruker, 1997 ▶); data reduction: *SAINT*; program(s) used to solve structure: *SHELXS97* (Sheldrick, 2008 ▶); program(s) used to refine structure: *SHELXL97* (Sheldrick, 2008 ▶); molecular graphics: *SHELXTL* (Sheldrick, 2008 ▶) and *PLATON* (Spek, 2003 ▶); software used to prepare material for publication: *SHELXTL*.

## Supplementary Material

Crystal structure: contains datablocks global, I. DOI: 10.1107/S1600536808040981/hk2582sup1.cif

Structure factors: contains datablocks I. DOI: 10.1107/S1600536808040981/hk2582Isup2.hkl

Additional supplementary materials:  crystallographic information; 3D view; checkCIF report

## Figures and Tables

**Table 1:** Hydrogen-bond geometry (Å, °)

*D*—H⋯*A*	*D*—H	H⋯*A*	*D*⋯*A*	*D*—H⋯*A*
O3—H3⋯O4	0.82	1.91	2.605 (2)	142
C5—H5⋯O1^i^	0.93	2.58	3.229 (2)	127
C8—H8⋯O3^ii^	0.93	2.56	3.481 (2)	170

Symmetry codes: (i) 
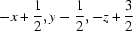
; (ii) 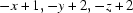.

**Fig. 1.**
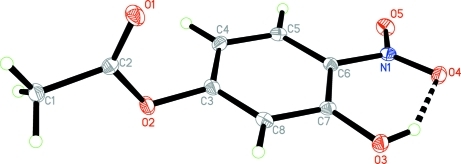
The molecular structure of the title molecule, with the atom-numbering scheme. Displacement ellipsoids are drawn at the 30% probability level. Hydrogen bond is shown as ashed line.

**Fig. 2.**
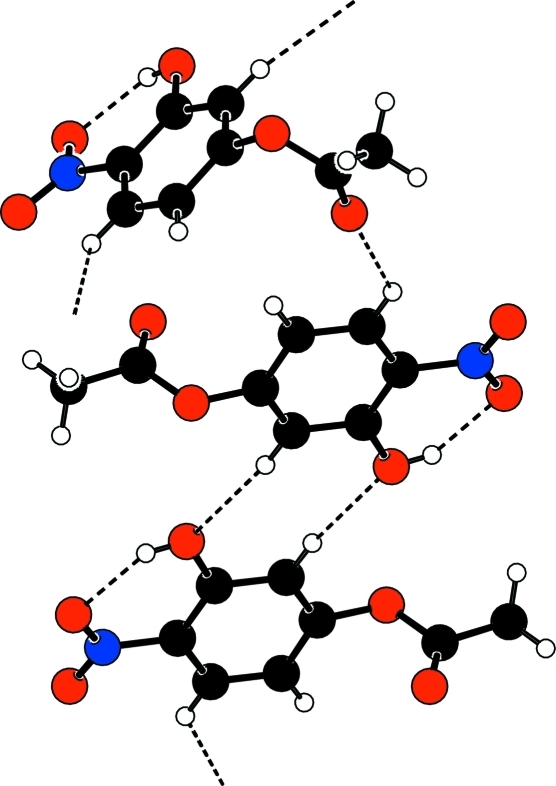
A partial packing diagram of the title compound. Hydrogen bonds are shown as dashed lines.

## supplementary crystallographic information

### Comment

Phenolic esters are useful intermediates in organic synthesis (Trollsås *et al.*, 1996; Svensson *et al.*, 1998; Atkinson *et al.*, 2005; Hu *et al.*, 2001). We have developed a new method for the syntheses of some phenolic esters (Ji *et al.*, 2006). The title compound is one of the products, and we report herein its crystal structure.

In the molecule of the title compound (Fig. 1) the bond lengths (Allen *et al.*,  1987) and angles are within normal ranges. The acetate group is oriented with respect to the aromatic ring at a dihedral angle of 85.30 (3)°. The intramolecular O-H···O hydrogen bond (Table 1) results in the formation of a nonplanar six-membered ring (N1/O3/O4/C6/C7/H3), adopting envelope conformation.

In the crystal structure, intermolecular C-H···O hydrogen bonds (Table 1) link the molecules (Fig. 2), in which they may be effective in the stabilization of the structure.

### Experimental

For the preparation of the title compound, 2-nitroresorcin acetate (239 mg, 1.0 mmol) was dissolved in chloroform (20 ml).  At 273-278 K, anhydrous AlCl_3_ (133.5 mg, 1 mmol) was added to this solution, the reaction was stirred at room temperature for 1 h, and then hydrochloric acid (5 ml, 10%) was added. The reaction mixture was extracted with chloroform and dried with anhydrous sodium sulfate. After concentration, the residue was separated by flash column chromatography and purified by recrystallization from chloroform (yield; 144 mg, 73%, m.p. 360 K). Spectroscopic analysis: IR (KBr, ν, cm^-1^): 3253, 3083, 2946, 1758, 1530, 1204, 1138, 978, 847. Analysis required for C_8_H_7_NO_5_: C 48.74; H  3.58; N 7.10%. Found: C 48.80; H 3.61; N 7.08%.

### Refinement

H atoms were positioned geometrically, with O-H = 0.82 Å (for OH) and C-H = 0.93 and 0.96 Å for aromatic and methyl H, respectively, and constrained to ride on their parent atoms with U_iso_(H) = xU_eq_(C,O), where x = 1.2 for aromatic H and x = 1.5 for all other H atoms.

### Crystal data

**Table:**

C_8_H_7_NO_5_	*F*(000) = 408
*M**_r_* = 197.15	*D*_x_ = 1.558 Mg m^−^^3^
Monoclinic, *P*2_1_/*n*	Melting point: 360 K
Hall symbol: -P 2yn	Mo *K*α radiation, λ = 0.71073 Å
*a* = 10.881 (2) Å	Cell parameters from 2650 reflections
*b* = 5.0543 (10) Å	θ = 2.2–27.5°
*c* = 15.318 (3) Å	µ = 0.13 mm^−^^1^
β = 93.75 (3)°	*T* = 153 K
*V* = 840.6 (3) Å^3^	Block, colorless
*Z* = 4	0.24 × 0.20 × 0.16 mm

### Data collection

**Table:**

Bruker P4 diffractometer	1449 independent reflections
Radiation source: fine-focus sealed tube	1232 reflections with *I* > 2σ(*I*)
graphite	*R*_int_ = 0.027
ω scans	θ_max_ = 25.0°, θ_min_ = 2.2°
Absorption correction: multi-scan (*SADABS*; Sheldrick, 1996)	*h* = −12→12
*T*_min_ = 0.969, *T*_max_ = 0.979	*k* = −6→5
5058 measured reflections	*l* = −18→15

### Refinement

**Table:**

Refinement on *F*^2^	Primary atom site location: structure-invariant direct methods
Least-squares matrix: full	Secondary atom site location: difference Fourier map
*R*[*F*^2^ > 2σ(*F*^2^)] = 0.028	Hydrogen site location: inferred from neighbouring sites
*wR*(*F*^2^) = 0.083	H-atom parameters constrained
*S* = 1.12	*w* = 1/[σ^2^(*F*_o_^2^) + (0.0475*P*)^2^ + 0.1223*P*] where *P* = (*F*_o_^2^ + 2*F*_c_^2^)/3
1449 reflections	(Δ/σ)_max_ = 0.001
129 parameters	Δρ_max_ = 0.18 e Å^−^^3^
0 restraints	Δρ_min_ = −0.20 e Å^−^^3^

### Special details

**Table:**

Geometry. All e.s.d.'s (except the e.s.d. in the dihedral angle between two l.s. planes) are estimated using the full covariance matrix. The cell e.s.d.'s are taken into account individually in the estimation of e.s.d.'s in distances, angles and torsion angles; correlations between e.s.d.'s in cell parameters are only used when they are defined by crystal symmetry. An approximate (isotropic) treatment of cell e.s.d.'s is used for estimating e.s.d.'s involving l.s. planes.
Refinement. Refinement of *F*^2^ against ALL reflections. The weighted *R*-factor *wR* and goodness of fit *S* are based on *F*^2^, conventional *R*-factors *R* are based on *F*, with *F* set to zero for negative *F*^2^. The threshold expression of *F*^2^ > σ(*F*^2^) is used only for calculating *R*-factors(gt) *etc*. and is not relevant to the choice of reflections for refinement. *R*-factors based on *F*^2^ are statistically about twice as large as those based on *F*, and *R*- factors based on ALL data will be even larger.

### Fractional atomic coordinates and isotropic or equivalent isotropic displacement parameters (Å^2^)

**Table:**

	*x*	*y*	*z*	*U*_iso_*/*U*_eq_	
O1	0.14195 (8)	0.58036 (19)	0.89666 (6)	0.0279 (3)	
O2	0.31702 (7)	0.35401 (18)	0.93249 (5)	0.0244 (3)	
O3	0.59843 (8)	1.04495 (18)	0.88687 (5)	0.0223 (2)	
H3	0.6350	1.1192	0.8486	0.033*	
O4	0.67226 (8)	1.08418 (17)	0.72904 (5)	0.0230 (2)	
O5	0.63078 (8)	0.77288 (18)	0.63478 (5)	0.0247 (2)	
N1	0.61633 (9)	0.8778 (2)	0.70551 (6)	0.0179 (3)	
C1	0.13908 (11)	0.2225 (3)	1.00085 (8)	0.0213 (3)	
H1A	0.0532	0.2620	1.0041	0.032*	
H1B	0.1807	0.2425	1.0577	0.032*	
H1C	0.1482	0.0438	0.9810	0.032*	
C2	0.19373 (11)	0.4079 (2)	0.93821 (7)	0.0179 (3)	
C3	0.38267 (10)	0.5030 (3)	0.87381 (8)	0.0195 (3)	
C4	0.38272 (11)	0.4184 (3)	0.78716 (8)	0.0207 (3)	
H4	0.3323	0.2804	0.7666	0.025*	
C5	0.46001 (11)	0.5463 (2)	0.73292 (8)	0.0194 (3)	
H5	0.4633	0.4919	0.6751	0.023*	
C6	0.53337 (10)	0.7573 (2)	0.76450 (7)	0.0164 (3)	
C7	0.53043 (10)	0.8455 (2)	0.85126 (7)	0.0168 (3)	
C8	0.45238 (10)	0.7116 (2)	0.90598 (7)	0.0189 (3)	
H8	0.4480	0.7643	0.9639	0.023*	

### Atomic displacement parameters (Å^2^)

**Table:**

	*U*^11^	*U*^22^	*U*^33^	*U*^12^	*U*^13^	*U*^23^
O1	0.0222 (5)	0.0277 (6)	0.0342 (5)	0.0061 (4)	0.0054 (4)	0.0129 (4)
O2	0.0180 (5)	0.0266 (5)	0.0290 (5)	0.0010 (4)	0.0041 (3)	0.0131 (4)
O3	0.0269 (5)	0.0225 (5)	0.0177 (4)	−0.0062 (4)	0.0019 (4)	−0.0023 (3)
O4	0.0233 (5)	0.0224 (5)	0.0232 (5)	−0.0057 (4)	0.0018 (3)	−0.0005 (4)
O5	0.0302 (5)	0.0282 (5)	0.0164 (4)	0.0030 (4)	0.0064 (4)	−0.0034 (4)
N1	0.0165 (5)	0.0200 (6)	0.0171 (5)	0.0032 (4)	0.0004 (4)	0.0005 (4)
C1	0.0219 (6)	0.0208 (7)	0.0216 (6)	−0.0008 (5)	0.0033 (5)	0.0030 (5)
C2	0.0176 (6)	0.0169 (6)	0.0190 (6)	−0.0003 (5)	0.0003 (5)	−0.0026 (5)
C3	0.0143 (6)	0.0208 (7)	0.0235 (6)	0.0042 (5)	0.0021 (5)	0.0071 (5)
C4	0.0177 (6)	0.0182 (7)	0.0255 (7)	−0.0008 (5)	−0.0033 (5)	0.0010 (5)
C5	0.0204 (6)	0.0191 (7)	0.0180 (6)	0.0029 (5)	−0.0022 (5)	−0.0012 (5)
C6	0.0150 (6)	0.0177 (6)	0.0166 (6)	0.0033 (5)	0.0012 (4)	0.0018 (4)
C7	0.0162 (6)	0.0158 (6)	0.0182 (6)	0.0035 (5)	−0.0016 (4)	−0.0003 (5)
C8	0.0197 (6)	0.0211 (7)	0.0159 (6)	0.0043 (5)	0.0022 (5)	0.0017 (5)

### Geometric parameters (Å, °)

**Table:**

O1—C2	1.1982 (15)	C1—H1C	0.9600
O2—C2	1.3773 (15)	C3—C8	1.3716 (18)
O2—C3	1.4032 (14)	C3—C4	1.3944 (17)
O3—C7	1.3447 (15)	C4—C5	1.3807 (17)
O3—H3	0.8200	C4—H4	0.9300
O4—N1	1.2489 (13)	C5—C6	1.3994 (17)
O5—N1	1.2255 (12)	C5—H5	0.9300
N1—C6	1.4521 (15)	C6—C7	1.4041 (16)
C1—C2	1.4923 (16)	C7—C8	1.4054 (17)
C1—H1A	0.9600	C8—H8	0.9300
C1—H1B	0.9600		
			
C2—O2—C3	118.26 (9)	C4—C3—O2	118.51 (11)
C7—O3—H3	109.5	C5—C4—C3	117.87 (12)
O5—N1—O4	121.88 (10)	C5—C4—H4	121.1
O5—N1—C6	119.32 (10)	C3—C4—H4	121.1
O4—N1—C6	118.79 (9)	C4—C5—C6	120.33 (11)
C2—C1—H1A	109.5	C4—C5—H5	119.8
C2—C1—H1B	109.5	C6—C5—H5	119.8
H1A—C1—H1B	109.5	C5—C6—C7	121.42 (11)
C2—C1—H1C	109.5	C5—C6—N1	117.92 (10)
H1A—C1—H1C	109.5	C7—C6—N1	120.63 (11)
H1B—C1—H1C	109.5	O3—C7—C6	125.16 (10)
O1—C2—O2	122.43 (11)	O3—C7—C8	117.20 (10)
O1—C2—C1	127.24 (11)	C6—C7—C8	117.62 (11)
O2—C2—C1	110.33 (10)	C3—C8—C7	119.85 (11)
C8—C3—C4	122.88 (11)	C3—C8—H8	120.1
C8—C3—O2	118.37 (10)	C7—C8—H8	120.1
			
C3—O2—C2—O1	−1.83 (17)	O5—N1—C6—C7	169.13 (10)
C3—O2—C2—C1	177.59 (10)	O4—N1—C6—C7	−9.92 (16)
C2—O2—C3—C8	98.78 (13)	C5—C6—C7—O3	179.46 (10)
C2—O2—C3—C4	−86.75 (14)	N1—C6—C7—O3	1.57 (18)
C8—C3—C4—C5	2.32 (18)	C5—C6—C7—C8	0.95 (17)
O2—C3—C4—C5	−171.88 (10)	N1—C6—C7—C8	−176.94 (10)
C3—C4—C5—C6	−1.34 (18)	C4—C3—C8—C7	−1.64 (18)
C4—C5—C6—C7	−0.25 (18)	O2—C3—C8—C7	172.57 (10)
C4—C5—C6—N1	177.70 (10)	O3—C7—C8—C3	−178.67 (10)
O5—N1—C6—C5	−8.83 (15)	C6—C7—C8—C3	−0.03 (17)
O4—N1—C6—C5	172.12 (9)		

### Hydrogen-bond geometry (Å, °)

**Table:**

*D*—H···*A*	*D*—H	H···*A*	*D*···*A*	*D*—H···*A*
O3—H3···O4	0.82	1.91	2.605 (2)	142
C5—H5···O1^i^	0.93	2.58	3.229 (2)	127
C8—H8···O3^ii^	0.93	2.56	3.481 (2)	170

Symmetry codes: (i) −*x*+1/2, *y*−1/2, −*z*+3/2; (ii) −*x*+1, −*y*+2, −*z*+2.

## References

[bb1] Allen, F. H., Kennard, O., Watson, D. G., Brammer, L., Orpen, A. G. & Taylor, R. (1987). *J. Chem. Soc. Perkin Trans. 2*, pp. S1–19.

[bb2] Atkinson, P. J., Bromidge, S. M., Duxon, M. S., Gaster, L. M., Hadley, M. S., Hammond, B., Johnson, C. N., Middlemiss, D. N., North, S. E., Price, G. W., Rami, H. K., Riley, G. J., Scott, C. M., Shaw, T. E., Starr, K. R., Stemp, G., Thewlis, K. M., Thomas, D. R., Thompson, M., Vong, A. K. K. & Watson, J. M. (2005). *Bioorg. Med. Chem. Lett.* **15**, 737–741. 10.1016/j.bmcl.2004.11.030 15664848

[bb3] Bruker (1997). *SMART *and *SAINT* . Bruker AXS Inc., Madison, Wisconsin, USA.

[bb4] Hu, B., Ellingboe, J., Gunawan, I., Han, S., Largis, E., Li, Z., Malamas, M., Mulvey, R., Oliphant, A., Sum, F.-W., Tillett, J. & Wong, V. (2001). *Bioorg. Med. Chem. Lett.* **11**, 757–760. 10.1016/s0960-894x(01)00063-4 11277513

[bb5] Ji, X. & Li, C. (2006). *Synthesis*, **15**, 2478–2482.

[bb6] Sheldrick, G. M. (1996). *SADABS* University of Göttingen, Germany.

[bb7] Sheldrick, G. M. (2008). *Acta Cryst.* A**64**, 112–122. 10.1107/S0108767307043930 18156677

[bb8] Spek, A. L. (2003). *J. Appl. Cryst.* **36**, 7–13.

[bb9] Svensson, M., Helgee, B., Skarp, K. & Andersson, G. (1998). *J. Mater. Chem.* **8**, 353–362.

[bb10] Trollsås, M., Orrenius, C., Sahlén, F., Gedde, U. W., Norin, T., Hult, A., Hermann, D., Rudquist, P., Komitov, L., Lagerwall, S. T. & Lindström, J. (1996). *J. Am. Chem. Soc.* **118**, 8542–8548.

